# Supplementary material for: The tumor-sentinel lymph node immuno-migratome reveals CCR7⁺ dendritic cells drive response to sequenced immunoradiotherapy
Source: Nat Commun. 2025 Jul 17;16:6578. doi: 10.1038/s41467-025-61780-4 (PMC12271439; doi:10.1038/s41467-025-61780-4)
Supplement: Supplementary file 1 — Supplementary Information [file 41467_2025_61780_MOESM1_ESM.pdf]

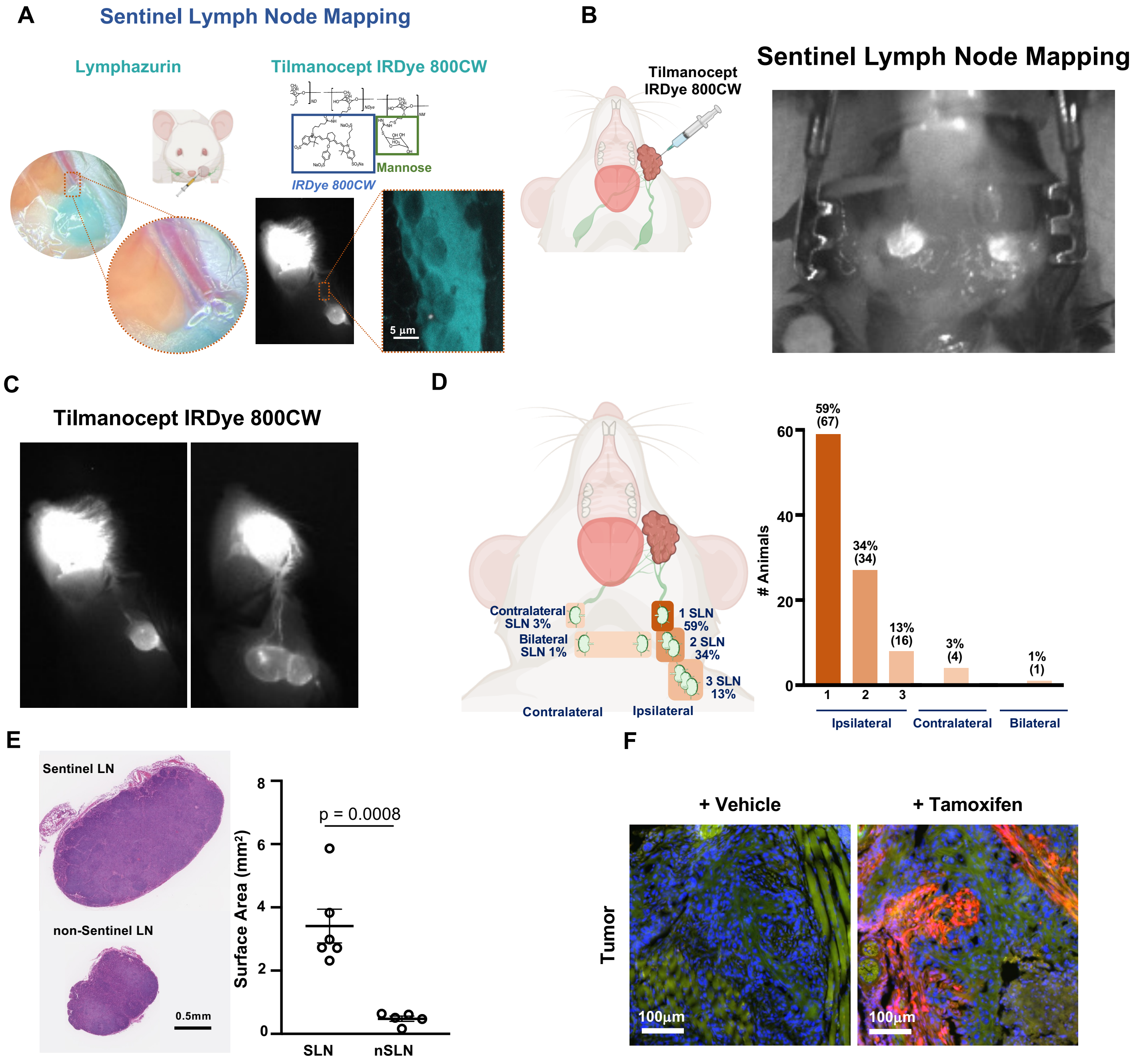

A
Sentinel Lymph Node Mapping
Lymphazurin
Tilmanocept IRDye 800CW
Mannose
IRDye 800CW
5 µm
B
Tilmanocept IRDye 800CW
Sentinel Lymph Node Mapping
C
Tilmanocept IRDye 800CW
D
Contralateral SLN 3%
Bilateral SLN 1%
1 SLN 59%
2 SLN 34%
3 SLN 13%
Contralateral
Ipsilateral
Animals
59% (67)
34% (34)
13% (16)
3% (4)
1% (1)
60
40
20
0
1
2
3
Ipsilateral
Contralateral
Bilateral
E
Sentinel LN
non-Sentinel LN
0.5mm
Surface Area (mm²)
p = 0.0008
8
6
4
2
0
SLN
nSLN
F
+ Vehicle
+ Tamoxifen
Tumor
100µm
100µm

Supplementary Figure 1: Host Antitumor Surveillance is Defined by a Diverse ImmunoMigratome to the Sentinel Lymph Node

(A) Sentinel lymph nodes (SLNs) were mapped using Lyphazurin and Tilmanocept IRDye 800CW. Lyphazurin, a blue dye, was injected submucosally into four adjacent sites of the oral cavity, enabling the visualization of the lymphatic channels. Tilmanocept IRDye 800CW, a mannose receptor-binding agent, provided high-resolution fluorescent imaging of the SLNs. Representative images show the SLN mapping with Lyphazurin and Tilmanocept IRDye 800CW, with high-magnification insets displaying dye tracking adjacent vasculature into the SLN and a microscopic photograph of a sentinel lymphatic channel after immunofluorescent staining with anti-LYVE. Representative images shown from >10 biologically independent mice; experiment repeated at least twice with similar results.

(B) Fluorescent imaging of SLNs in tumor-bearing mice using Tilmanocept IRDye 800CW. The representative image confirms the localization of the dye within the lymphatic vessels and SLNs. Representative images shown from >10 biologically independent mice; experiment repeated at least twice with similar results.

(C) High-resolution fluorescent imaging with Tilmanocept IRDye 800CW demonstrates precise mapping of SLNs. Representative images with distinct patterns of SLNs in tumor-bearing animals. Representative images shown from >10 biologically independent mice; experiment repeated at least twice with similar results.

(D) **(*Left*)** Cartoon representation of patterns of SLN in tumor-bearing animals with **(*right*)** quantification shown. 59% (n=67) exhibited ipsilateral SLN localization, 34% (n=34) contralateral, and 13% (n=16) bilateral localization. Representative images shown from >10 biologically independent mice; experiment repeated at least twice with similar results.

(E) Quantitative analysis of SLN and non-SLN surface area. H&E-stained sections reveal significant differences in the surface area, confirming effective SLN mapping (n = 5-6/group; p = 0.0008). Data are presented as mean values +/- SEM; p values calculated by two-sided unpaired Student's t-test. Representative images shown from 5 biologically independent mice; experiment repeated at least twice with similar results.

(F) Representative immunofluorescence imaging of tumors from vehicle- and tamoxifen-treated R26-CreERT2 x Ai9 reporter mice, showing tdTomato+ cell labeling in the tumor 72 hours after intraoral tamoxifen injection, demonstrating effective Cre activation and tdTomato labeling in the tumor; supports Main Figure 1B and 1C. Representative images shown from 3 biologically independent mice; experiment repeated at least twice with similar results.

Created in BioRender. Saddawi-Konefka, R. (2025) https://BioRender.com/emood6a, https://BioRender.com/m675tdd. Source data are provided as a Source Data file.

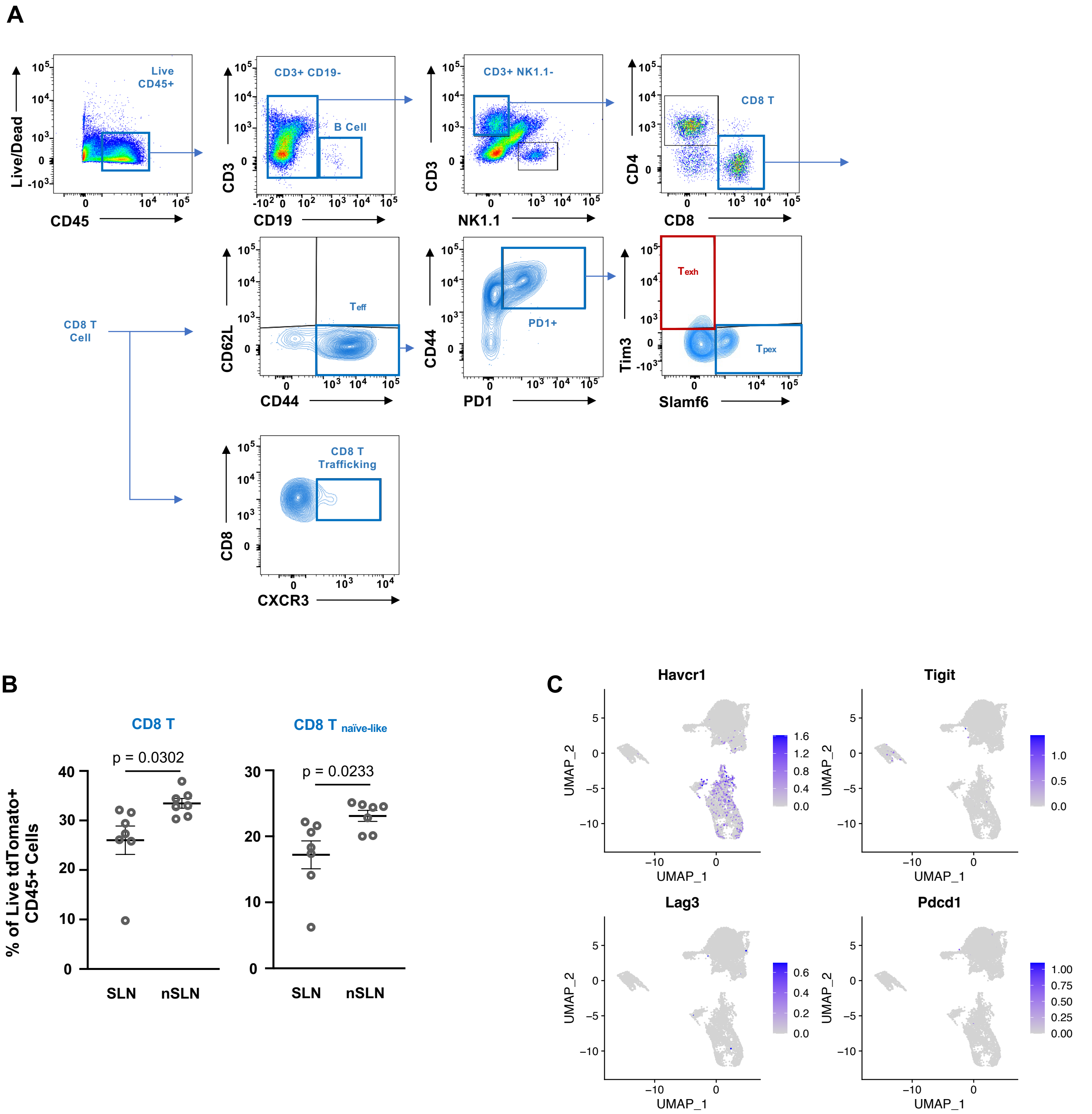

A
Live CD45+
Live/Dead
CD45
CD3+ CD19-
B Cell
CD3
CD19
CD3+ NK1.1-
NK1.1
CD8 T
CD4
CD8
CD8 T Cell
Teff
CD62L
CD44
PD1+
PD1
Texh
Tpex
Tim3
Slamf6
CD8 T Trafficking
CXCR3
B
CD8 T
CD8 T naïve-like
p = 0.0302
p = 0.0233
% of Live tdTomato+ CD45+ Cells
SLN
nSLN
C
Havcr1
Tigit
Lag3
Pdcd1
UMAP_1
UMAP_2

Supplementary Figure 2: Functional Profiling of the Tumor-to-SLN Immune Migratome Reveals Compartmental Differences and Lack of Regulatory B Cell Signatures

(A) Flow cytometric gating strategy, supporting Figure 1E-F and Supp Fig 1I.

(B) Quantification of tdTomato+ CD8+ T cells and CD8+ naïve-like T cells in SLNs versus nSLNs 72 hours after intratumoral tamoxifen injection in 4MOSC1 tumor-bearing R26-CreERT2 x Ai9 reporter mice, revealing a higher relative abundance of total T cells that are naïve-like in nSLN versus SLN; supports main Figure 1F (n=7/group; experiment repeated at least twice with similar results). Data are presented as mean values +/- SEM; p values calculated by two-sided unpaired Student's t-test.

(C) UMAP feature plots showing expression of canonical regulatory B cell-associated genes (Havcr1, Tigit, Lag3, Pdcd1) within the tdTomato+ B cell cluster, demonstrating the absence of distinct regulatory B cell subset phenotypes in the SLN immune migratome[1]; supports main Fig 1I. n=2 biologically independent samples/group

Source data are provided as a Source Data file.

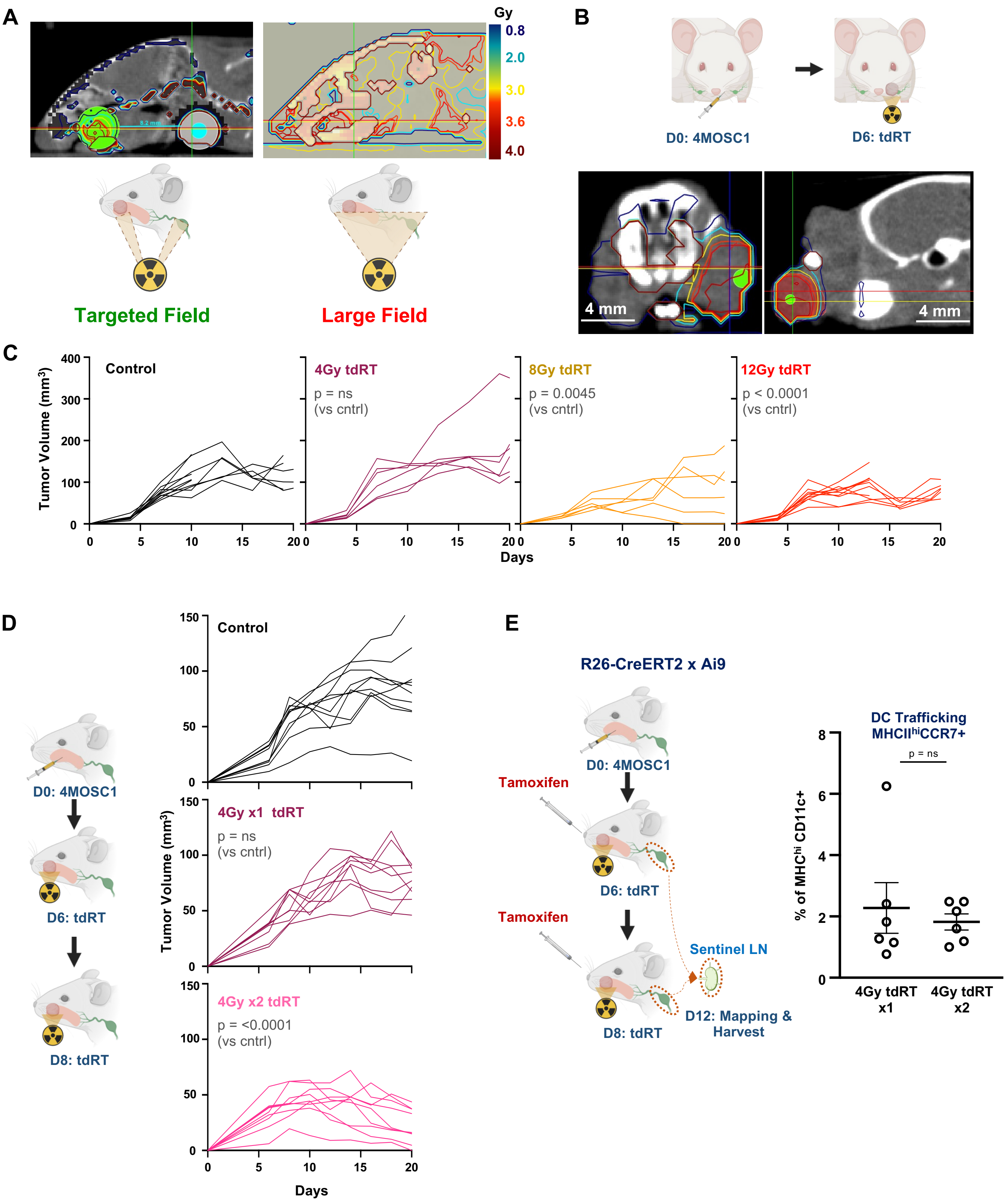
A
Gy
0.8
2.0
3.0
3.6
4.0
Targeted Field
Large Field
B
D0: 4MOSC1
D6: tdRT
4 mm
4 mm
C
Tumor Volume (mm³)
Control
4Gy tdRT
p = ns
(vs cntrl)
8Gy tdRT
p = 0.0045
(vs cntrl)
12Gy tdRT
p < 0.0001
(vs cntrl)
Days
D
D0: 4MOSC1
D6: tdRT
D8: tdRT
Control
4Gy x1 tdRT
p = ns
(vs cntrl)
4Gy x2 tdRT
p = <0.0001
(vs cntrl)
Tumor Volume (mm³)
Days
E
R26-CreERT2 x Ai9
D0: 4MOSC1
Tamoxifen
D6: tdRT
Tamoxifen
Sentinel LN
D8: tdRT
D12: Mapping & Harvest
DC Trafficking
MHCIIhiCCR7+
p = ns
% of MHChi CD11c+
4Gy tdRT x1
4Gy tdRT x2

Supplementary Figure 3. Tumor-Directed Radiation Promotes Local Immunosurveillance in the Tumor Microenvironment Without Cytotoxicity

(A) ***(top)*** Representative CT images of sagittal series overlaid with radiation planning. ***(bottom)*** Cartoon schema of targeted field radiation therapy versus large field radiation therapy. Representative of n=10 biologically independent samples; experiment was independently repeated at least twice with similar results.

(B) ***(top)*** Cartoon schema of the experimental approach. WT animals injected with 4MOSC1 into the buccal space and then treated with tdRT on day 6. ***(bottom)*** Representative CT images of coronal and sagittal series overlaid with tumor-targeted radiation planning. Representative of n=10 biologically independent samples; experiment was independently repeated at least twice with similar results.

(C) Tumor growth curves for control and tdRT-treated groups (4Gy, 8Gy, 12Gy tdRT). Tumor volumes were measured over time, with significant differences observed at specific time points (n = 7 mice per group, p = ns for 4Gy, p = 0.0045 for 8Gy, p < 0.0001 for 12Gy). Best-fit lines and p values calculated by simple linear regression (two-sided).

(D) (***left***) Cartoon schema illustrating treatment regimens for tumor-directed radiation therapy (tdRT) with either a single fraction (4 Gy × 1, Day 6) or two fractions (4 Gy × 2, Day 6 and Day 8) in 4MOSC1 tumor-bearing animals. (***right***) Tumor growth curves for control and tdRT-treated groups. Tumor volumes were measured over time (n = 10 mice per group; p = ns for 4Gy × 1, p < 0.0001 for 4Gy × 2). Best-fit lines and p values calculated by simple linear regression (two-sided).

(E) (***left***) Cartoon schema illustrating experimental design for assessing migratory dendritic cell trafficking in response to fractionated tdRT. R26-CreERT2 x Ai9 mice were orthotopically implanted with 4MOSC1 tumors and received either one or two 4 Gy fractions of tdRT on Days 6 and 8, along with intratumoral tamoxifen on Days 6 and 8. Sentinel lymph nodes were harvested on Day 12 for flow cytometric analysis. (***right***) Quantification of MHCII+ CCR7+ migratory dendritic cells (DCs) within CD11c+ populations in SLNs reveals no significant difference between single-dose and two-dose tdRT groups (n = 6 mice per group, p = ns). Data are presented as mean values +/- SEM; p values calculated by two-sided unpaired Student’s t-test. Best-fit lines and p values calculated by simple linear regression (two-sided).

Created in BioRender. Saddawi-Konefka, R. (2025) https://BioRender.com/m675tdd. Source data are provided as a Source Data file.

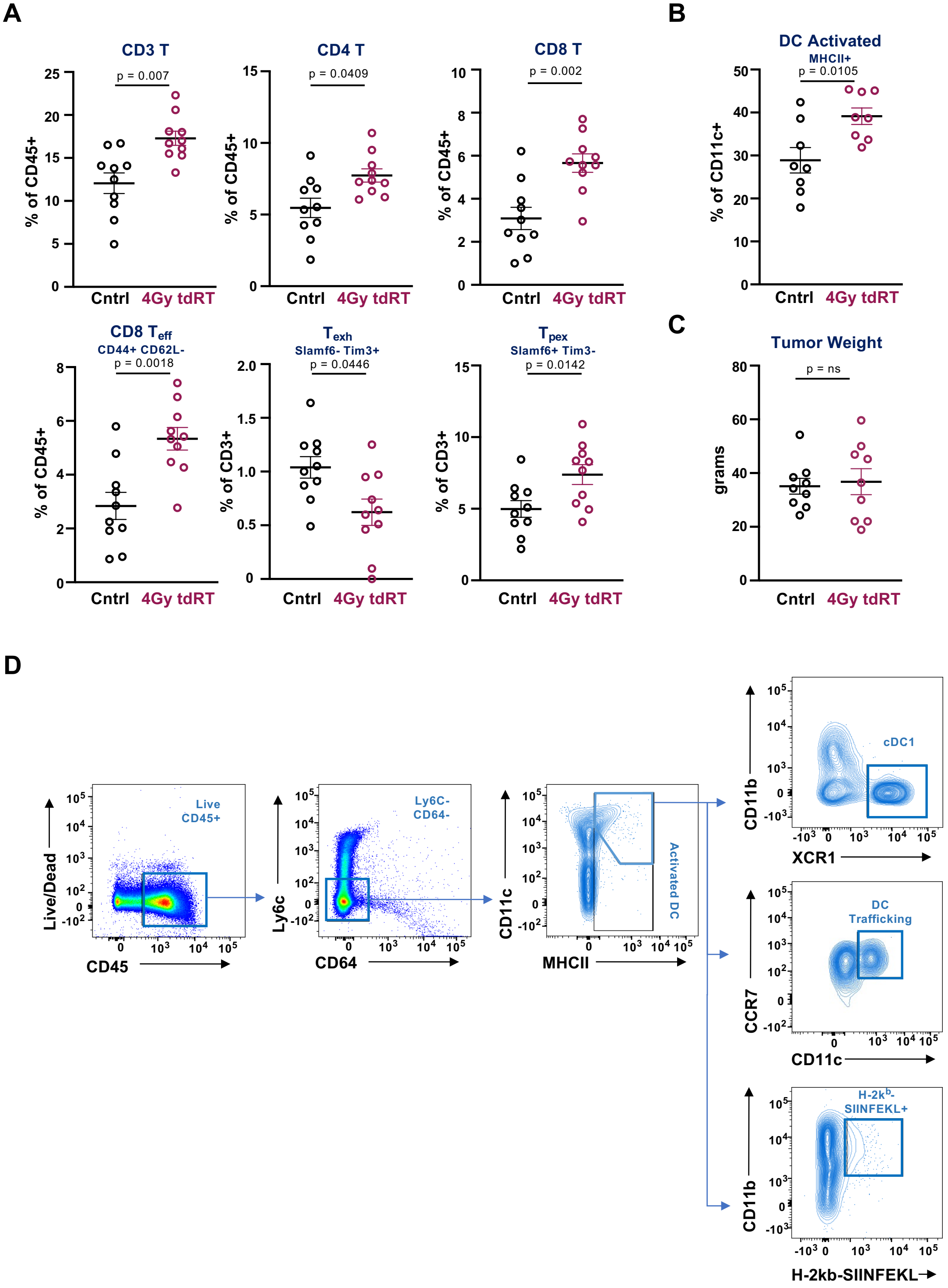
A
CD3 T
p = 0.007
% of CD45+
Cntrl
4Gy tdRT
CD4 T
p = 0.0409
CD8 T
p = 0.002
B
DC Activated
MHCII+
p = 0.0105
% of CD11c+
CD8 Teff
CD44+ CD62L-
p = 0.0018
Texh
Slamf6- Tim3+
p = 0.0446
% of CD3+
Tpex
Slamf6+ Tim3-
p = 0.0142
C
Tumor Weight
p = ns
grams
D
Live/Dead
CD45
Live CD45+
Ly6c
CD64
Ly6C- CD64-
CD11c
MHCII
Activated DC
CD11b
XCR1
cDC1
CCR7
CD11c
DC Trafficking
CD11b
H-2kb-SIINFEKL
H-2kb-SIINFEKL+

Supplementary Figure 4. Tumor-Directed Radiation Enhances Effector Immune Infiltration and Dendritic Cell Activation in the Tumor Microenvironment

(A) Flow cytometric analysis of tumor-infiltrating lymphocyte populations post-tdRT. Quantification of the percentage of parent population is shown (n = 10 mice per group, p = 0.007 for CD3 T cells, p = 0.0409 for CD4 T cells, p = 0.002 for CD8 T cells, p = 0.0018 for CD8 Teff, p = 0.0446 for Texh, p = 0.0142 for Tpex). Data are presented as mean values +/- SEM; p values calculated by two-sided unpaired Student's t-test.

(B) Flow cytometric analysis of tumor-infiltrating activated $MHCII^{hi}$ dendritic cells post-tdRT. Quantification of the percentage of parent population is shown (n = 8 mice per group, p = 0.0105 for DCs).

(C) Tumor weight in grams, supporting Figure 2 E (n = 9-10 mice per group, p = ns).

(D) Flow cytometric gating strategy, supporting Figure 2F-H.

Source data are provided as a Source Data file.

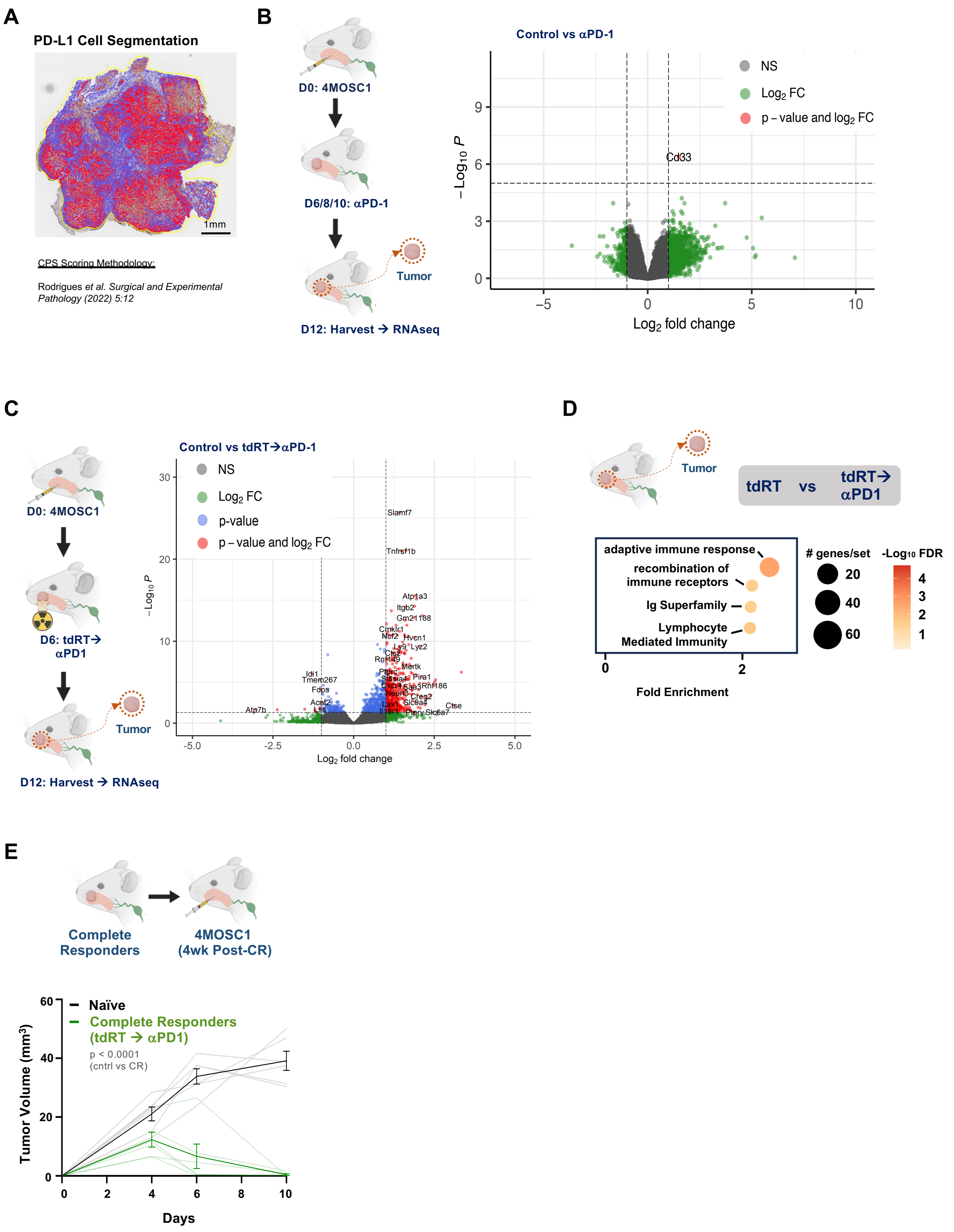

A
PD-L1 Cell Segmentation
1mm
CPS Scoring Methodology:
Rodrigues et al. Surgical and Experimental Pathology (2022) 5:12
B
D0: 4MOSC1
D6/8/10: αPD-1
Tumor
D12: Harvest → RNAseq
Control vs αPD-1
NS
Log2 FC
p – value and log2 FC
Cd33
– Log10 P
Log2 fold change
C
D0: 4MOSC1
D6: tdRT→ αPD1
Tumor
D12: Harvest → RNAseq
Control vs tdRT→αPD-1
NS
Log2 FC
p-value
p – value and log2 FC
Slamf7
Tnfrsf1b
Atp1a3
Itgb2
Gm21188
Cmklr1
Ncf2
Hvcn1
Ly9
Lyz2
Ctss
Rnf149
Mertk
Pira1
Idi1
Tmem267
Fdps
Acat2
Lss
Atp7b
Ctse
Log2 fold change
–Log10 P
D
Tumor
tdRT vs tdRT→ αPD1
adaptive immune response
recombination of immune receptors
Ig Superfamily
Lymphocyte Mediated Immunity
Fold Enrichment
genes/set
-Log10 FDR
E
Complete Responders
4MOSC1 (4wk Post-CR)
Naïve
Complete Responders (tdRT → αPD1)
p < 0.0001 (cntrl vs CR)
Tumor Volume (mm3)
Days

Supplementary Figure 5. Tumor-Directed Radiation Upregulates Programs of Antitumor Immune Surveillance to Potentiate the αPD-1 ICI Tumor Response

(A) PD-L1 cell segmentation. **(top)** Representative image showing PD-L1 cell segmentation. Combined Positive Score (CPS) scoring methodology referenced from Rodrigues et al., Surgical and Experimental Pathology (2022) 5:12. Representative of n=4 biologically independent samples; experiment was independently repeated at least twice with similar results.

(B) Volcano plot of differentially expressed genes in tumors following αPD-1 ICI treatment, highlighting genes involved in immune responses.

(C) Volcano plot of differentially expressed genes in tumors following tdRT and αPD-1 ICI treatment, highlighting genes involved in immune responses, supporting main Figure 3C.

(D) Pathway enrichment analysis comparing tumors treated with tdRT alone versus tdRT→αPD-1 combination therapy, highlighting pathways associated with adaptive immunity and lymphocyte-mediated responses. X-axis represents gene sets ranked by normalized enrichment score (NES); Y-axis represents the $-\log_{10}$(FDR q-value).

(E) **(top)** Cartoon schema of the experimental approach showing 4MOSC1 complete responders post tdRT + αPD-1 ICI. **(bottom)** Tumor volume measurements in naïve and complete responders (tdRT→αPD-1) (n = 5-6 mice per group, $p < 0.0001$). Best-fit lines and p values calculated by simple linear regression (two-sided).

Created in BioRender. Saddawi-Konefka, R. (2025) https://BioRender.com/m675tdd. Source data are provided as a Source Data file.

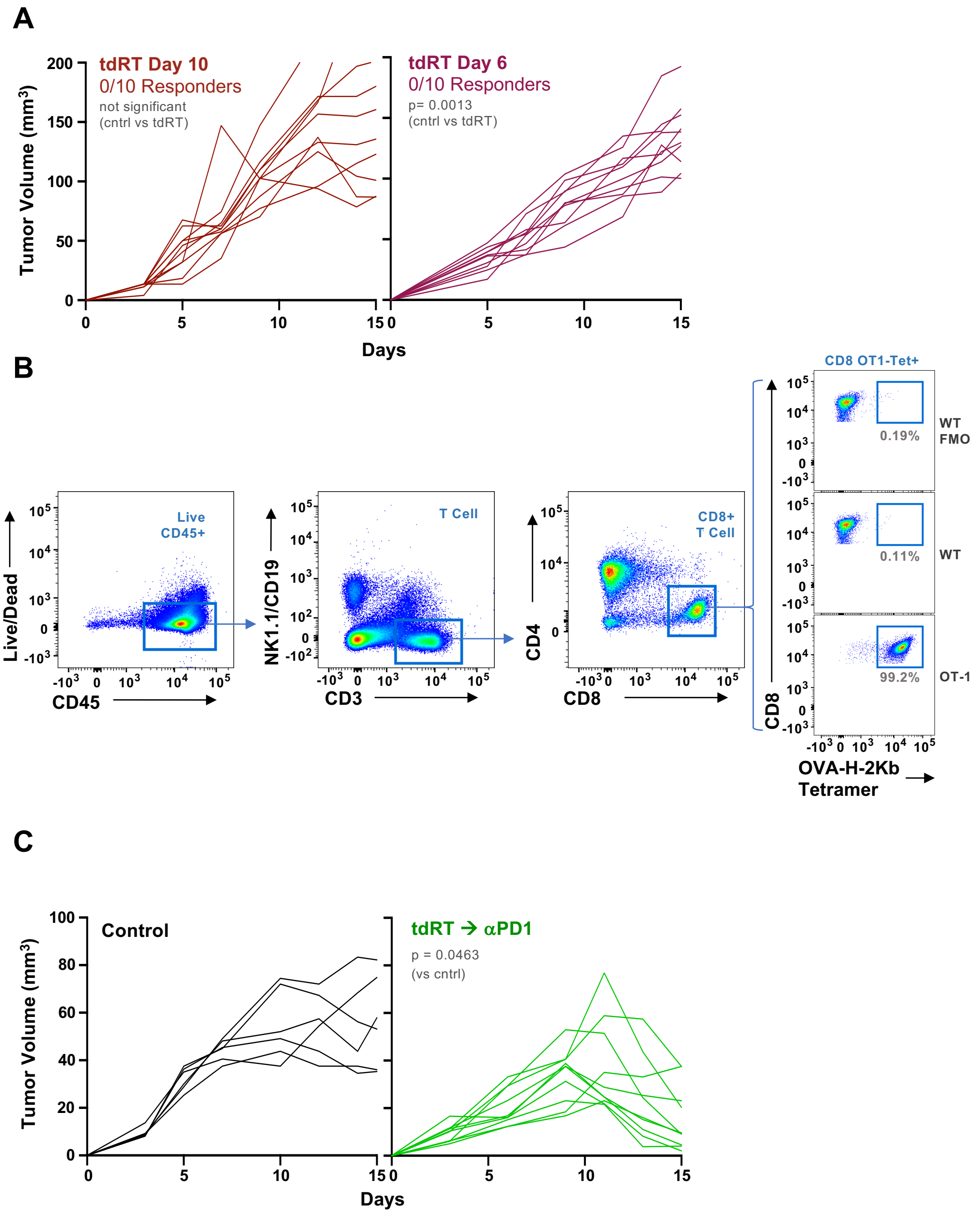
A
tdRT Day 10
0/10 Responders
not significant
(cntrl vs tdRT)
tdRT Day 6
0/10 Responders
p= 0.0013
(cntrl vs tdRT)
Tumor Volume (mm³)
Days
B
Live/Dead
CD45
Live CD45+
NK1.1/CD19
CD3
T Cell
CD4
CD8
CD8+ T Cell
CD8 OT1-Tet+
0.19%
WT FMO
0.11%
WT
99.2%
OT-1
OVA-H-2Kb Tetramer
C
Control
tdRT → αPD1
p = 0.0463
(vs cntrl)
Tumor Volume (mm³)
Days

Supplementary Figure 6. The Host Response to Tumor-Directed Immunoradiotherapy is Coordinated in Regional Lymphatics

(A) Tumor growth curves for monotherapy with tdRT delivered on day 10 or day 6 treatment groups (n=10 mice per group, p = ns day 10 tdRT or p = 0.0013 day 6 tdRT compared to control), supporting Figure 4A-B. Best-fit lines and p values calculated by simple linear regression (two-sided).

(B) Flow cytometric gating strategy, supporting Figure 4C.

(C) Tumor growth curves for control and tdRT→αPD-1 treatment groups (n=10 mice per group, p = 0.0463), supporting Figure 4I.

Source data are provided as a Source Data file.

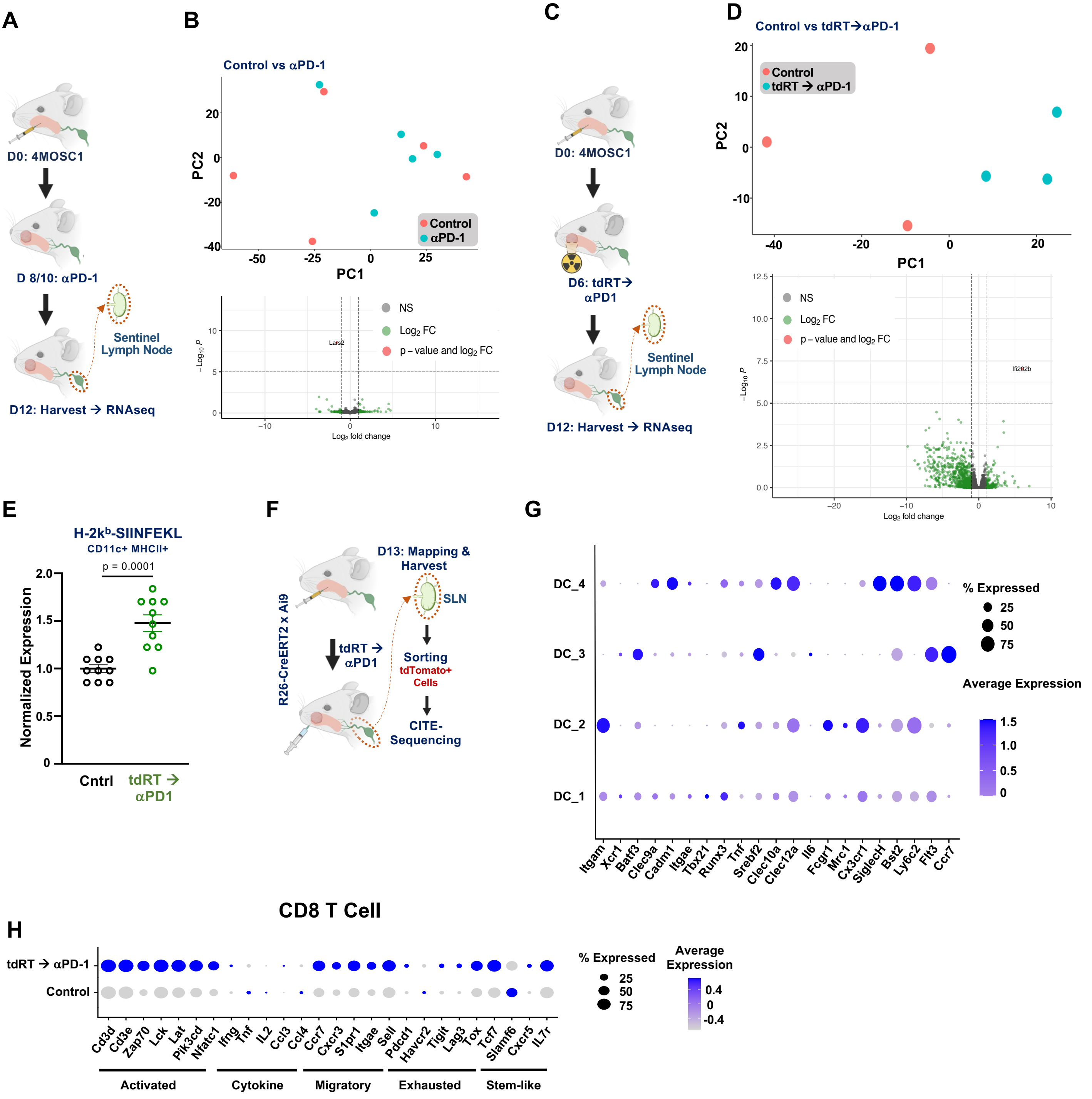
A
D0: 4MOSC1
D 8/10: αPD-1
Sentinel Lymph Node
D12: Harvest → RNAseq
B
Control vs αPD-1
PC2
PC1
Control
αPD-1
NS
Log2 FC
p – value and log2 FC
Lars2
Log2 fold change
C
D0: 4MOSC1
D6: tdRT→ αPD1
Sentinel Lymph Node
D12: Harvest → RNAseq
D
Control vs tdRT→αPD-1
Control
tdRT → αPD-1
Ifi202b
E
H-2kb-SIINFEKL
CD11c+ MHCII+
p = 0.0001
Normalized Expression
Cntrl
tdRT → αPD1
F
R26-CreERT2 x Ai9
D13: Mapping & Harvest
SLN
tdRT → αPD1
Sorting
tdTomato+ Cells
CITE-Sequencing
G
DC_4
DC_3
DC_2
DC_1
% Expressed
Average Expression
Itgam Xcr1 Batf3 Clec9a Cadm1 Itgae Tbx21 Runx3 Tnf Srebf2 Clec10a Clec12a Il6 Fcgr1 Mrc1 Cx3cr1 SiglecH Bst2 Ly6c2 Flt3 Ccr7
CD8 T Cell
H
tdRT → αPD-1
Control
% Expressed
Average Expression
Cd3d Cd3e Zap70 Lck Lat Pik3cd Nfatc1 Ifng Tnf IL2 Ccl3 Ccl4 Ccr7 Cxcr3 S1pr1 Itgae Sell Pdcd1 Havcr2 Tigit Lag3 Tox Tcf7 Slamf6 Cxcr5 IL7r
Activated
Cytokine
Migratory
Exhausted
Stem-like

Supplementary Figure 7. Tumor-Directed Immunoradiotherapy Enhances Dendritic Cell Antitumor Surveillance Across the Tumor and Sentinel Lymph Node

(A) Cartoon schema of the experimental approach. WT animals injected with 4MOSC1 tumors were treated with αPD-1 on Day 8 &10 and then subjected to sentinel lymph node (SLN) mapping. RNA sequencing from the sentinel lymph node showing normalized enrichment scores for various immune response pathways post-treatment. Data support Figure 5A.

(B) (**top**) Principal component analysis (PCA) of the sentinel lymph node from 4MOSC1-tumor bearing animals treated with αPD-1 monotherapy; (**bottom**) Volcano plot of differentially expressed genes in tumors following αPD-1 ICI treatment, highlighting genes involved in immune responses.

(C) Cartoon schema of the experimental approach. WT animals injected with 4MOSC1 tumors were treated with tdRT on Day 6 and then subjected to sentinel lymph node (SLN) mapping. RNA sequencing from the sentinel lymph node showing normalized enrichment scores for various immune response pathways post-treatment. Data support Figure 5A.

(D) (**top**) Principal component analysis (PCA) of the sentinel lymph node from 4MOSC1-tumor bearing animals treated with tdRT monotherapy; (**bottom**) Volcano plot of differentially expressed genes in tumors following αPD-1 ICI treatment, highlighting genes involved in immune responses.

(E) Flow cytometric analysis of H-2Kb-SIINFEKL expression on MHCII+ CD11c+ cells in the sentinel lymph node post-treatment (n=10 mice per group, p = 0.0001). Data are presented as mean values +/- SEM; p values calculated by two-sided unpaired Student's t-test.

(F) Cartoon schema of the experimental approach. ROSA-26 x Ai9 animals were injected with 4MOSC1 tumors were treated with tdRT→αPD-1, labeled with tamoxifen in the tumor and then subjected to sentinel lymph node (SLN) mapping. Sorted live tdTomato+ cells from the SLN were then sent for CITE-sequencing. CITE-sequencing was performed on sorted tdTomato+ cells isolated from the SLNs, as described in the methods.

(G) Dot plot analysis of dendritic cell (DC) populations after CITE-sequencing, showing the percentage of expression and average expression of various markers in DC_1, DC_2, DC_3, and DC_4 populations, supporting Figure 5G. n=2 biologically independent samples/group

(H) Dot plot analysis of CD8 T cell populations after CITE-sequencing, showing the percentage of expression and average expression of various markers in comparing control to sequenced therapy with tdRT→αPD-1. n=2 biologically independent samples/group

Created in BioRender. Saddawi-Konefka, R. (2025) https://BioRender.com/m675tdd. Source data are provided as a Source Data file.

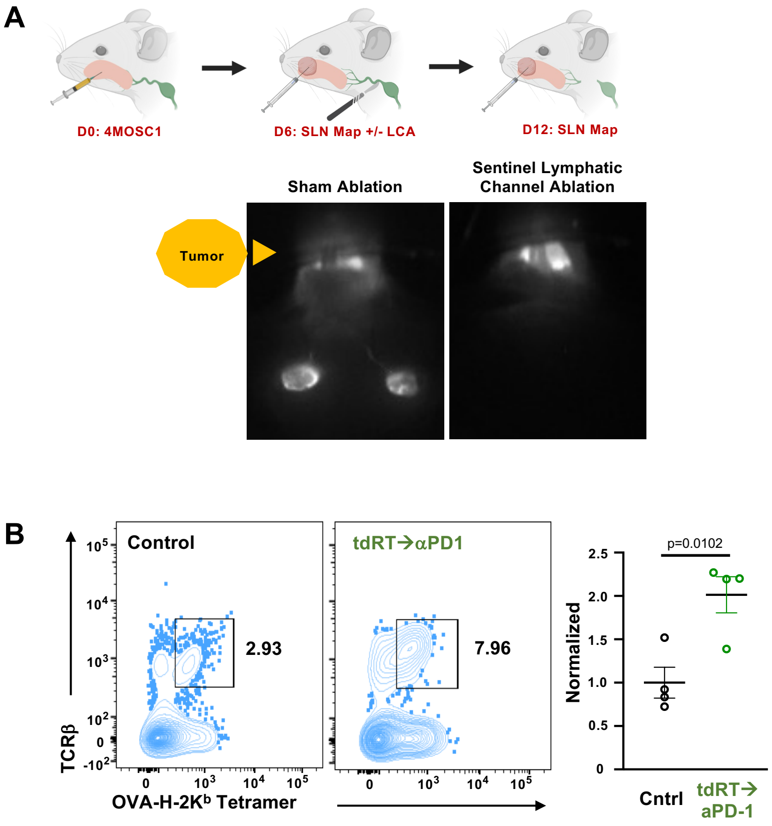
A
D0: 4MOSC1
D6: SLN Map +/- LCA
D12: SLN Map
Sham Ablation
Sentinel Lymphatic Channel Ablation
Tumor
B
Control
tdRT→αPD1
2.93
7.96
TCRβ
OVA-H-2Kb Tetramer
p=0.0102
Normalized
Cntrl
tdRT→ aPD-1

Supplementary Figure 8. CCR7+ Dendritic Cell Trafficking and MMP9-Dependent Entry into the Sentinel Lymph Node are Critical for Immunoradiotherapy Efficacy

(A) (top) Cartoon schema of the experimental approach. WT animals injected with 4MOSC1 tumors subjected to sentinel lymph node lymphatic channel ablation (SLN LCA) or sham ablation, followed by tdRT→αPD-1 treatment and subsequent SLN mapping. (bottom) Representative images of sham ablation and sentinel lymphatic channel ablation. Representative of n=6 biologically independent samples; experiment was independently repeated at least twice with similar results.

(B) Flow cytometric analysis of Ovalbumin-specific T cells (4MOSC1-LucOS model) in the SLN, comparing control and tdRT→αPD-1 treated groups. Quantification of normalized percentages is shown (n=5 mice per group, p=0.0102), supporting Figure 6C. Data are presented as mean values +/- SEM; p values calculated by two-sided unpaired Student's t-test.

Created in BioRender. Saddawi-Konefka, R. (2025) https://BioRender.com/m675tdd. Source data are provided as a Source Data file.

1 Bod, L. *et al.* B-cell-specific checkpoint molecules that regulate anti-tumour immunity. *Nature* **619**, 348-356 (2023). https://doi.org/10.1038/s41586-023-06231-0
