# Supplementary material for: The tumor-sentinel lymph node immuno-migratome reveals CCR7⁺ dendritic cells drive response to sequenced immunoradiotherapy
Source: Nat Commun. 2025 Jul 17;16:6578. doi: 10.1038/s41467-025-61780-4 (PMC12271439; doi:10.1038/s41467-025-61780-4)
Supplement: Supplementary file 2 — Reporting Summary [file 41467_2025_61780_MOESM2_ESM.pdf]

nature portfolio

Corresponding author(s): Robert Saddawi-Konefka, Joseph Califano

Last updated by author(s): 4/28/2025

Reporting Summary

Nature Portfolio wishes to improve the reproducibility of the work that we publish. This form provides structure for consistency and transparency in reporting. For further information on Nature Portfolio policies, see our Editorial Policies and the Editorial Policy Checklist.

Statistics

For all statistical analyses, confirm that the following items are present in the figure legend, table legend, main text, or Methods section.

n/a	Confirmed	
☐	☒	The exact sample size (*n*) for each experimental group/condition, given as a discrete number and unit of measurement
☐	☒	A statement on whether measurements were taken from distinct samples or whether the same sample was measured repeatedly
☐	☒	The statistical test(s) used AND whether they are one- or two-sided<br>*Only common tests should be described solely by name; describe more complex techniques in the Methods section.*
☐	☒	A description of all covariates tested
☐	☒	A description of any assumptions or corrections, such as tests of normality and adjustment for multiple comparisons
☐	☒	A full description of the statistical parameters including central tendency (e.g. means) or other basic estimates (e.g. regression coefficient) AND variation (e.g. standard deviation) or associated estimates of uncertainty (e.g. confidence intervals)
☐	☒	For null hypothesis testing, the test statistic (e.g. F, t, r) with confidence intervals, effect sizes, degrees of freedom and P value noted<br>*Give P values as exact values whenever suitable.*
☒	☐	For Bayesian analysis, information on the choice of priors and Markov chain Monte Carlo settings
☐	☒	For hierarchical and complex designs, identification of the appropriate level for tests and full reporting of outcomes
☒	☐	Estimates of effect sizes (e.g. Cohen's *d*, Pearson's *r*), indicating how they were calculated

Our web collection on statistics for biologists contains articles on many of the points above.

Software and code

Policy information about availability of computer code

Data collection	Data collection was performed using standard software for flow cytometry (e.g., BD LSR II Fortessa software), imaging (e.g., Zeiss Axioscan Z1 software for slide scanning), and animal positioning for radiotherapy (e.g., SmART-Plan version 1.3.1). No custom software was used for data collection.
Data analysis	Data analysis involved GraphPad Prism version 9 for statistical analyses, QuPath version 0.2.3 for image analysis, and Cell Ranger v7.0.1 for single-cell RNA sequencing alignment and quantification. Additional analyses, including differential expression, were performed using DESeq2 (v1.32.0), and single-cell analysis was conducted with Seurat (v4). No custom code was developed for this study.

For manuscripts utilizing custom algorithms or software that are central to the research but not yet described in published literature, software must be made available to editors and reviewers. We strongly encourage code deposition in a community repository (e.g. GitHub). See the Nature Portfolio guidelines for submitting code & software for further information.

Data

Policy information about availability of data

All manuscripts must include a data availability statement. This statement should provide the following information, where applicable:

- Accession codes, unique identifiers, or web links for publicly available datasets
- A description of any restrictions on data availability
- For clinical datasets or third party data, please ensure that the statement adheres to our policy

Bulk RNAsequencing data generated in this study are available in the Sequence Read Archive (SRA) under BioProject accession number PRJNA1183332.

BioProject PRJNA1183332 can be accessed at https://dataview.ncbi.nlm.nih.gov/object/PRJNA1183332?reviewer=mu9ou9jusl82k8j4o3fkl3ruua.

Additionally, CITEsequencing and TCRsequencing datasets are currently under private access; reviewers can access them using the secure tokens provided.

GSE276437 can be accessed at https://www.ncbi.nlm.nih.gov/geo/query/acc.cgi?acc=GSE276437 with the token uvcrkomshtyxrif.
GSE276434 can be accessed at https://www.ncbi.nlm.nih.gov/geo/query/acc.cgi?acc=GSE276434 with the token ujobgsmgvboxtox.

Upon publication, these datasets, including the bulk-RNA sequencing data featured in the manuscript, will be made publicly accessible without restriction.

Research involving human participants, their data, or biological material

Policy information about studies with human participants or human data. See also policy information about sex, gender (identity/presentation), and sexual orientation and race, ethnicity and racism.

Reporting on sex and gender	not applicable
Reporting on race, ethnicity, or other socially relevant groupings	not applicable
Population characteristics	not applicable
Recruitment	not applicable
Ethics oversight	not applicable

Note that full information on the approval of the study protocol must also be provided in the manuscript.

Field-specific reporting

Please select the one below that is the best fit for your research. If you are not sure, read the appropriate sections before making your selection.

[x] Life sciences [ ] Behavioural & social sciences [ ] Ecological, evolutionary & environmental sciences

For a reference copy of the document with all sections, see nature.com/documents/nr-reporting-summary-flat.pdf

Life sciences study design

All studies must disclose on these points even when the disclosure is negative.

Sample size	Sample size was determined based on prior studies and statistical power calculations to ensure sufficient power to detect significant effects.
Data exclusions	No data exclusions were performed; all data points were included in the analysis.
Replication	All experiments were replicated at least twice with consistent results, ensuring reproducibility.
Randomization	Mice were randomly assigned to experimental groups to control for potential confounding variables.
Blinding	Investigators were blinded to group allocation during data collection and analysis to minimize bias.

Reporting for specific materials, systems and methods

We require information from authors about some types of materials, experimental systems and methods used in many studies. Here, indicate whether each material, system or method listed is relevant to your study. If you are not sure if a list item applies to your research, read the appropriate section before selecting a response.

Materials & experimental systems

n/a	Involved in the study	
[ ]	[x]	Antibodies
[ ]	[x]	Eukaryotic cell lines
[x]	[ ]	Palaeontology and archaeology
[ ]	[x]	Animals and other organisms
[x]	[ ]	Clinical data
[x]	[ ]	Dual use research of concern
[x]	[ ]	Plants

Methods

n/a	Involved in the study	
[x]	[ ]	ChIP-seq
[ ]	[x]	Flow cytometry
[x]	[ ]	MRI-based neuroimaging

Antibodies

Antibodies used	CD45 (clone 30-F11, BioLegend, catalog #100714), CD3 (clone 17A2, BioLegend, catalog #100203), CD8a (clone 53-6.7, BioLegend, catalog #100714), CD4 (clone RM4-4, BioLegend, catalog #100418), Slamf6 (clone 330AJ, BioLegend, catalog #134009), PD-1 (clone 29 F.1A12, BioLegend, catalog #135205), CD44 (clone IM7, BioLegend, catalog #103036), CD19 (clone 6D5, BioLegend, catalog #115506), CXCR3 (clone S18001A, BioLegend, catalog #126515), Tim3 (clone RMT3-23, BioLegend, catalog #119723), NK1.1 (clone PK136, BioLegend, catalog #108705), CD69 (clone H1.2F3, BioLegend, catalog #104506), CD62L (clone MEL-14, BioLegend, catalog #104406), BST2 (clone 129C1, BioLegend, catalog #127001), Ly6C (clone HK1.4, BioLegend, catalog #128031), CD11b (clone M1/70, BioLegend, catalog #101215), CD11c (clone N418, BioLegend, catalog #117310), Siglec H (clone 551, BioLegend, catalog #129703), XCR1 (clone ZET, BioLegend, catalog #148205), CD64 (clone X54-5/7.1, BioLegend, catalog #139303), CD103 (clone 2E7, BioLegend, catalog #121413), SIRPa (clone P84, BioLegend, catalog #144006), MHCII (clone M5/114.15.2, BioLegend, catalog #107616), CD80 (clone 16-10A1, BioLegend, catalog #104723), CD86 (clone GL-1, BioLegend, catalog #105005), Ep-CAM (clone G8.8, BioLegend, catalog #118206), H-2Kb-SIINFEKL (clone 25-D1.16, BioLegend, catalog #141603), IL-2 (clone JES6-5H4, BioLegend, catalog #503803), IFNγ (clone XMG1.2, BioLegend, catalog #505806), αCD8 (BioXCell, clone YTS169.4, used for CD8 depletion at 250 mg/mouse per dose)
Validation	All primary antibodies were validated by the manufacturer for use in murine samples and the specified applications (e.g., flow cytometry, immunofluorescence). Validation data and application-specific information are available on the respective suppliers' websites (BioLegend, BD Biosciences). Where applicable, validation was further confirmed in this study by specificity in gating strategies and consistency with expected cell populations, as shown in supplementary figures. Additional antibodies targeting specific epitopes, such as H-2Kb-SIINFEKL, were validated through consistent antigen-specific responses. Additional validation was conducted by assessing expected depletion or inhibition effects in vivo, such as CD8+ T-cell depletion using αCD8 and inhibition of lymphocyte egress using FTY720, as described in our methods and shown in supplementary figures. Detailed validation data can be found on suppliers' websites, including BioXCell and Sigma-Aldrich, for blocking and depleting antibodies.

Eukaryotic cell lines

Policy information about cell lines and Sex and Gender in Research

Cell line source(s)	The 4MOSC1 syngeneic mouse HNSCC cell line was developed by the Gutkind lab and characterized in a prior study (Nature Communications, 2019) line was designed to model tobacco-associated mutational landscapes relevant to head and neck cancer.
Authentication	Cell lines were authenticated through STR profiling by the original developer and confirmed based on consistent morphology and growth characteristics under experimental conditions.
Mycoplasma contamination	All cell lines were tested for mycoplasma contamination and confirmed negative before use in experiments.
Commonly misidentified lines (See ICLAC register)	No commonly misidentified cell lines were used in this study.

Animals and other research organisms

Policy information about studies involving animals; ARRIVE guidelines recommended for reporting animal research, and Sex and Gender in Research

Laboratory animals	C57BL/6 mice, aged 6-8 weeks, were used in all experiments. Mice were sourced from Jackson Laboratory and maintained under specific pathogen-free conditions.
Wild animals	This study did not involve wild animals.
Reporting on sex	Both male and female mice were used in the study. Data were disaggregated by sex where relevant, and sex was considered during study design to evaluate any potential sex-based differences. No significant sex-based differences were observed; therefore, combined data are presented.
Field-collected samples	This study did not involve field-collected samples.
Ethics oversight	All animal procedures were approved by the Institutional Animal Care and Use Committee (IACUC) at the University of California, San Diego (UCSD) under protocol number S16200, with Principal Investigator Dr. Joseph Califano. The protocol, titled 'Mouse model for cancer development and drug treatment of cancer,' was approved on 08/23/2022 and is valid until 07/21/2025, covering the use of Mus musculus (mouse) in compliance with federal and institutional regulations.

Note that full information on the approval of the study protocol must also be provided in the manuscript.

Plants

Seed stocks	Not applicable; no plant material was used in this study.
Novel plant genotypes	not applicable
Authentication	not applicable

Flow Cytometry

Plots

Confirm that:

- [x] The axis labels state the marker and fluorochrome used (e.g. CD4-FITC).
- [x] The axis scales are clearly visible. Include numbers along axes only for bottom left plot of group (a 'group' is an analysis of identical markers).
- [x] All plots are contour plots with outliers or pseudocolor plots.
- [x] A numerical value for number of cells or percentage (with statistics) is provided.

Methodology

Sample preparation	Cells were isolated from [tissue type, e.g., lymph nodes, tumors] of C57BL/6 mice and prepared using standard protocols. Samples were processed by [e.g., mechanical dissociation, enzymatic digestion] to create single-cell suspensions, followed by washing and staining with fluorochrome-conjugated antibodies.
Instrument	Flow cytometry data were collected using a BD LSR II Fortessa (BD Biosciences), configured with the appropriate lasers for detecting the selected fluorochromes.
Software	FlowJo version X (Tree Star) was used for data analysis and visualization. Data files were exported as FCS files and analyzed according to the gating strategies.
Cell population abundance	The abundance of cell populations was quantified by calculating the percentage of gated cells relative to the total viable cells, with statistics on cell count or percentage provided in the figures.
Gating strategy	Gating was performed based on forward scatter (FSC) and side scatter (SSC) properties to exclude debris, followed by sequential gating on live cells and specific markers for each cell type. Boundaries between positive and negative populations were defined based on fluorescence minus one (FMO) controls. An example of the gating strategy is provided in the supplementary information.

- [x] Tick this box to confirm that a figure exemplifying the gating strategy is provided in the Supplementary Information.
